# Activity of factor XII‐Locarno

**DOI:** 10.1002/rth2.12054

**Published:** 2017-11-13

**Authors:** Bassem M. Mohammed, Ivan Ivanov, Anton Matafonov, Jonas Emsley, David Gailani

**Affiliations:** ^1^ Department of Pathology Microbiology and Immunology Vanderbilt University Medical Center Nashville TN USA; ^2^ Department of Clinical Pharmacy Faculty of Pharmacy Cairo University Cairo Egypt; ^3^ Department of Bioengineering and Organic Chemistry Tomsk Polytechnic University Tomsk Russia; ^4^ Centre for Biomolecular Sciences School of Pharmacy University of Nottingham Nottingham UK

**Keywords:** factor XI, factor XII, point mutation, polyphosphate, prekallikrein

## Abstract

**Essentials**

- Conversion of FXII to α‐FXIIa on surfaces requires cleavage after Arginine 353.
- Replacing Arg353 with alanine results in a single chain form (FXII‐R353A) that has some activity.
- Replacing Arg‐353 with proline (FXII‐Locarno, FXII‐R353P) reduces activity of single chain FXII compared to FXII‐R353A.
- Proper conformation of the FXII activation loop is required for single chain FXII activity.

**Background:**

Factor XII (FXII) Locarno is a natural variant with proline replacing Arg353 at the activation cleavage site, preventing conversion to the fully active protease factor XIIa (FXIIa). Recently, we showed that FXII restricted to a single chain form (sc‐FXII) by replacing Arg353 with alanine expresses proteolytic activity that is enhanced by cofactors such as polyphosphate.

**Objective:**

To determine if the Pro353 substitution affects the activity of sc‐FXII.

**Methods:**

Wild type FXII (FXII‐WT), FXII‐R353A, and FXII Locarno (FXII‐R353P) were tested for their abilities to activate prekallikrein, and to induce thrombin generation and coagulation in plasma in a factor XI‐dependent manner.

**Results:**

FXII‐WT is converted to FXIIa by autoactivation in the presence of polyphosphate, and by incubation with kallikrein. FXII‐R353P and FXII‐R353A were not converted to FXIIa by these methods. Despite this, FXII‐R353A converts prekallikrein to kallikrein, and the reaction is enhanced by polyphosphate. FXII‐R353P also converts prekallikrein to kallikrein, but at a slower rate than FXII‐R353A. In FXII‐deficient plasma induced to clot with silica, FXII‐R353A is a better promoter of factor XI‐dependent thrombin generation and coagulation than FXII‐R353P.

**Conclusion:**

The activity of sc‐FXII is sensitive to perturbations in the activation loop, which contains residue 353. Homology modeling based on the crystal structure of the FXII homolog tissue plasminogen activator suggests that Pro353 introduces changes in the shape and flexibility of the activation loop that disrupt key interactions that support an active conformation in sc‐FXII.

## 1 INTRODUCTION

Exposure of blood to a variety of biological and non‐biological substances or surfaces initiates contact activation. Central to this process is the plasma protein factor XII (FXII), the precursor of the serine protease α‐factor XIIa (α‐FXIIa).^1^ In blood plasma, α‐FXIIa activates the kallikrein‐kinin system by converting plasma prekallikrein (PK) to α‐kallikrein,^2^ and initiates coagulation by activating factor XI (FXI).^3^ It is well‐recognized that FXII undergoes autocatalysis to α‐FXIIa by cleavage after Arg353 upon surface binding,^4^ however, the mechanism that initiates this process has been debated. Recently, we showed that FXII restricted to the single‐chain precursor form (sc‐FXII) by replacement of Arg353 with alanine still expresses proteolytic activity toward FXII, PK, and FXI in the presence of anionic cofactors such as polyphosphate (Poly‐P).^5^ While the activity of sc‐FXII is orders of magnitude lower than that of α‐FXIIa, it would provide a triggering mechanism for contact activation.

FXII‐Locarno is a cross‐reactive material positive FXII variant that contains proline in place of Arg353 (FXII‐R353P). It was identified in a patient with a prolonged activated partial thromboplastin time (aPTT) and no bleeding symptoms.^6^, ^7^ Like FXII‐R353A, FXII‐R353P cannot be converted to α‐FXIIa, and was initially thought to lack proteolytic activity.^6^, ^7^ We compared the activities of FXII containing FXII‐R353A and FXII‐R353P, to determine if the non‐conservative proline substitution at the activation cleavage site altered the activity of sc‐FXII.

## 2 METHODS

### 2.1 Materials

Pooled normal plasma (PNP), Precision Biologic. FXII‐deficient plasma (FXII‐dp), George King. FXII, PK, α‐kallikrein high molecular weight kinnogen (HK) and corn trypsin inhibitor (CTI), Enzyme Research Lab (South Bend, IN, USA). PTT‐A, Diagnostica Stago (Parsippany, NJ, USA). S‐2302 (H‐D‐prolyl‐L‐phenylalanyl‐L‐arginine‐p‐nitroaniline), DiaPharma (West Chester, OH, USA). Size‐fractionated Poly‐P (60‐200 phosphate groups, not calcium saturated) was a gift from Dr. Thomas Renné (Karolinska Institute). Poly‐P activity is completely destroyed by acid hydrolysis.^8^ Antibodies to FXI (O1A6),^9^ FXIIa (559C‐X181‐D06 [D06]),^10^ and kallikrein (559A‐M202‐H03 [H03]) are described.^10^, ^11^ Goat polyclonal IgG against human FXII, Affinity Biologicals (Ancaster, ON, Canada).

### 2.2 Recombinant proteins

**Figure 1:**
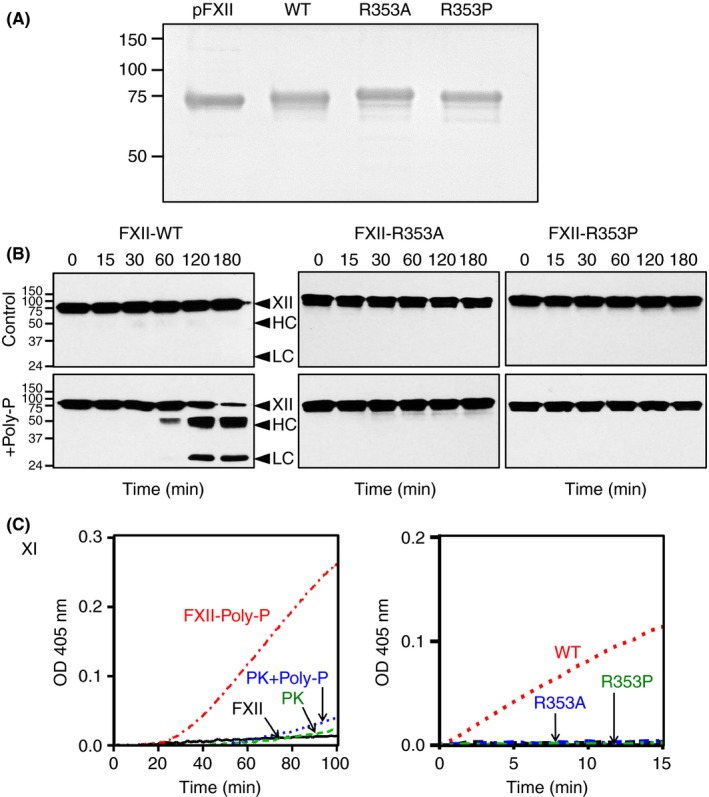
FXII activation. (A) Recombinant FXII. Coomassie blue‐stained non‐reducing SDS‐PAGE of plasma FXII (pFXII) and recombinant FXII‐wild type (WT), FXII‐R353A, and FXII‐R353P (~1.5 μg per lane). (B) Two‐hundred nanomolar FXII‐WT, FXII‐R353A or FXII‐R353P incubated at 37°C in assay buffer in the absence (top panels) or presence (bottom panels) of 70 μmol L^−1^ Poly‐P. At indicated time points (minutes, at top), samples were removed into reducing SDS sample buffer, size‐fractionated by SDS‐PAGE gel, blotted onto nitrocellulose membranes, and probed with goat anti‐FXII polyclonal antibody. Arrows to the right of blots for WT‐FXII show positions of controls for sc‐FXII (XII) and the heavy chain (HC) and light chain (LC) of α‐FXIIa. Positions of molecular mass standards (in kilodaltons) are shown on the left. (C) Left Panel*:* 200 nmol L^−1^ FXII‐WT or 200 nmol L^−1^ PK‐WT was incubated at 37°C in assay buffer supplemented with 200 μmol L^−1^ S‐2302 in the presence or absence of 70 μmol L^−1^ Poly‐P. Changes in OD at 405 nm over time were monitored. Right Panel: FXII‐WT, FXII‐R353A or FXII‐R353P (200 nmol L^−1^) was incubated with α‐Kallikrein (20 nmol L^−1^) at 37°C in assay buffer for 1 hour then supplemented with anti‐kallikrein antibody (H03) (20 nmol L^−1^) for 10 minute. Reaction mixtures were supplemented with S‐2302 (200 μmol L^−1^) and changes in OD 405 nm were monitored at 37°C for 15 minute

FXII^12^ or PK^13^ cDNAs were expressed in HEK293 cells under serum free conditions, and purified by anion exchange chromatography (Figure 1A) as reported.^5^ cDNAs for FXII‐R353A and FXII‐R353P were prepared by site‐directed mutagenesis.

### 2.3 Western blots

FXII (200 nmol L^−1^) in Assay Buffer (20 mmol L^−1^ HEPES, 100 mmol L^−1^ NaCl, 0.1% PEG‐8000, 10 μmol L^−1^ ZnCl_2_) was incubated at 37°C with or without 70 μmol L^−1^ Poly‐P (total phosphate group concentration). At various times, aliquots were removed into reducing SDS‐sample buffer, size fractionated by SDS‐PAGE and evaluated by western blot using goat anti‐human FXIl IgG. Detection was with HRP conjugated rabbit anti‐goat IgG and chemiluminescence.

### 2.4 Chromogenic assays

Reactions were run in Assay Buffer in 96‐well plates coated with PEG‐20,000. FXII (200 nmol L^−1^) was incubated with α‐kallikrein (20 nmol L^−1^) at 37°C. After 1 hour H03 (40 nmol L^−1^) and S‐2302 (200 μmol L^−1^) were added. Changes in OD 405 nm were monitored on a microplate reader. FXII (200 nmol L^−1^) autoactivation, and reciprocal FXII (200 nmol L^−1^) and PK (200 nmol L^−1^) activation, with or without 70 μmol L^−1^ Poly‐P, were monitored by following S‐2302 cleavage.

### 2.5 Thrombin generation

Thrombin generation was measured in plasma as described.^14^, ^15^ FXII‐dp (80 μL) containing 415 μmol L^−1^ Z‐Gly‐Gly‐Arg‐AMC (Bachem, Torrance, CA, USA) was reconstituted with FXII (400 nmol L^−1^), and anti‐FXI IgG O1A6 (20 μg mL^−1^) or vehicle. Contact activation was initiated by adding PTT‐A reagent (16% v/v final concentration). Thrombin generation was initiated by adding 10 μL of 20 mmol L^−1^ HEPES pH 7.4, 100 mmol L^−1^ CaCl_2_, 6% BSA and fluorescence was monitored over 60 min on a Fluoroskan Ascent fluorometer, and converted to thrombin generated using the manufacturers software. Assays were performed in triplicate. Endogenous thrombin potential (ETP‐area under the curve, reported in nm.min) was determined using GraphPad Prism software.

### 2.6 Plasma clotting assays

aPTT assays were performed on a STart4 Coagulation Analyzer (Diagnostica Stago). PNP or FXII‐dp supplemented with vehicle or FXII were tested, using PTT‐A reagent to trigger contact activation. Assays were performed in triplicate.

## 3 RESULTS AND DISCUSSION

FXII‐Locarno (FXII‐R353P) is not converted to α‐FXIIa because proline replaces arginine at the activation cleavage site (Arg353‐Val354 bond).^6^, ^7^ We compared it to the previously described FXII‐R353A to determine the effects of Pro353 on the intrinsic activity of sc‐FXII.^5^ Wild type FXII (FXII‐WT) undergoes autocatalysis to α‐FXIIa in the presence of Poly‐P (Figure 1B) resulting in increased capacity to cleave the substrate S‐2302 (Figure 1C).^5^ FXII‐R353A and FXII‐R353P are not cleaved (Figure 1B), and do not have increased amidolytic activity under similar conditions. During contact activation α‐kallikrein converts FXII to α‐FXIIa by cleavage after Arg353. As expected, neither FXII‐R353A nor FXII‐R353P are converted to α‐FXIIa by α‐kallikrein (Figure 1D).

**Figure 2:**
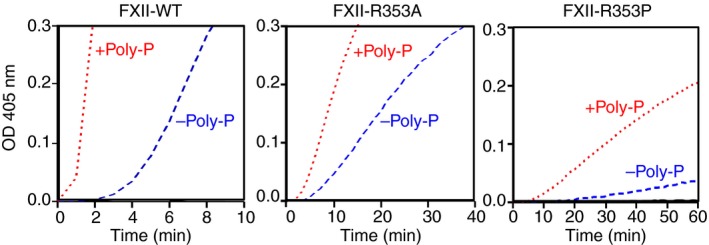
Poly‐P enhances FXII‐dependent PK activation. Two hundred nanomolaar FXII‐WT (left panel), FXII‐R353A (middle panel) or FXII‐R353P (right panel) was incubated with 200 nmol L^−1^ PK in assay buffer supplemented with S‐2302 (200 μmol L^−1^) at 37°C in the presence (red lines) or absence (blue lines) of 70 μmol L^−1^ Poly‐P. Changes in OD 405 were monitored continuously over time. Note the differences in time scales between the panels. Curves shown are representative examples of reactions run in triplicate

When FXII and PK are mixed in buffer, reciprocal conversion to α‐FXIIa and α‐kallikrein occurs by a reaction enhanced by Poly‐P (Figure 2, left).^5^, ^10^ PK undergoes little autocatalysis in the presence of Poly‐P (Figure 1C),^5^ and is dependent on FXII to convert it to α‐kallikrein. As reported, FXII‐R353A catalyzes PK conversion to α‐kallikrein (Figure 2, middle),^5^ demonstrating the proteolytic activity of sc‐FXII. FXII‐R353P also catalyzes PK activation, but at a lower rate than FXII‐R353A (Figure 2, right), suggesting structural changes introduced by Pro353 compromise activity. PK circulates as a complex with the cofactor HK, which enhances FXIIa‐mediated PK activation on surfaces.^16^ HK did not enhance PK activation by FXII‐R353A or FXII‐R353P with Poly‐P (data not shown).

**Figure 3:**
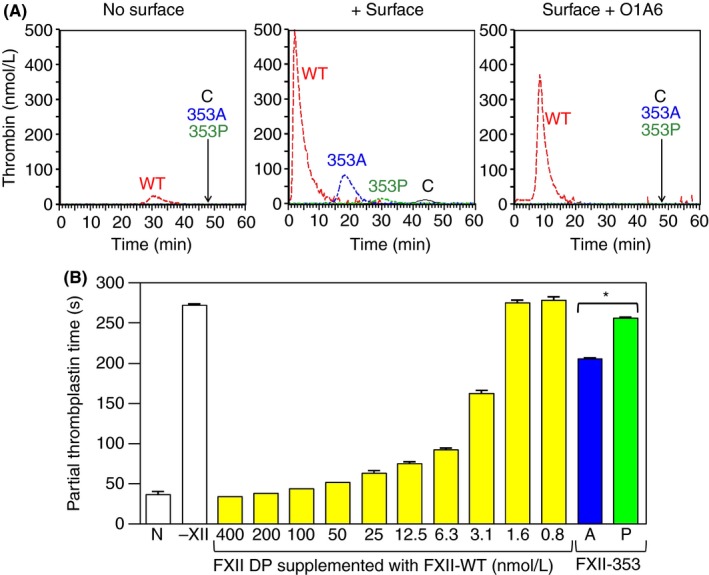
Plasma thrombin generation and clotting assays. (A) Thrombin generation in FXII‐deficient plasma supplemented with 400 nmol L^−1^ FXII‐WT (red), FXII‐R353A (blue), FXII‐R353P (green) or in the absence of FXII (black). Reactions were run in the absence of surface (Left panel) or with 16% PTT‐A (middle and right panel). Reactions in the right panel were run in the presence of FXI inhibitor antibody O1A6. Curves shown are averages of triplicate runs provided by the Thrombinoscope^®^ software. Absolute values and standard deviations for peak thrombin generation and ETP are given in the text. (B) PTT‐A initiated plasma clotting times (aPTTs). White bars show average aPTTs for PNP and FXII‐dp plasma. Yellow bars are average aPTTs for FXII‐dp supplemented with varying concentrations of FXII‐WT (0.78 to 400 nmol L^−1^). The blue and green bars are FXII‐dp supplemented with 400 nmol L^−1^ FXII‐R353A or FXII‐R353P, respectively. Results are presented as mean of three separate runs and the error bars represent standard deviations. *P < .05

α‐FXIIa promotes thrombin generation in plasma by activating FXI, a homolog of PK.^3^ We assessed the capacity of FXII to activate FXI using thrombin generation assays. Little thrombin generation occurs when FXII species are added to FXII‐dp in the absence of an inducer of contact activation (Figure 3A, left). The small peak produced by FXII‐WT probably reflects a trace of FXIIa in the FXII‐WT preparation. Adding a silica‐based reagent to plasma containing FXII‐WT promotes robust thrombin generation (Figure 3A, middle, peak 499 ± 2 nmol L^−1^; ETP 1909 ± 4 nmol L^−1^ min^−1^). FXII‐R353A also supports surface‐induced thrombin generation, but with reduced activity compared to FXII‐WT (peak 82 ± 4 nmol L^−1^; ETP 559 ± 19 nm min^−1^). The difference between FXII‐WT and FXII‐R353A likely reflects conversion of the former to α‐FXIIa. FXII‐R353P had a smaller effect on thrombin generation than FXII‐R353A (peak 15 ± 1 nmol L^−1^; ETP 143 ± 2 nmol L^−1^ min^−1^) that was only slightly greater than negative control (peak 8 ± 5 nmol L^−1^; ETP 62 ± 39 nmol L^−1^ min^−1^). An anti‐FXI antibody blocked the effects of FXII‐R353A and FXII‐R353P, and reduced and delayed the effects of FXII‐WT (Figure 3A, right), consistent with FXII promoting thrombin generation by activating FXI.

We compared FXI‐WT, FXII‐R353A, FXII‐R353P in aPTT assays, which also require FXI activation by contact activation. In Figure 3B, aPTTs of PNP (36.4 ± 2.1 seconds) and FXII‐dp (271 ± 5.1 seconds) are shown as white bars, while yellow bars show the effects of FXII‐WT on the aPTT of FXII‐dp, up to the normal plasma FXII concentration (400 nmol L^−1^). FXII‐R353A and FXII‐R353P shorten the aPTT of FXII‐dp slightly, with effects equivalent to <1% of the activity of FXII‐WT. This supports the premise that it is α‐FXIIa that is primarily responsible for FXI activation in normal plasma in the aPTT. The effect of FXII‐R353A was significantly greater than FXII‐R353P (206.2 ± 6.1 vs 256.5 ± 3.3, respectively, *P *<* *.05). Taken as a whole, the data with PK activation and thrombin generation assays, and the aPTT data, suggest that proline at position 353 in FXII has a deleterious effect on sc‐FXII activity.

Activation of serine protease zymogens typically involves internal proteolysis within an activation loop, creating a free N‐terminus for the catalytic domain that stabilizes the protease S1 specificity pocket. In human FXII this cleavage is after Arg353. Recently, we showed that forms of FXII that are not cleaved after Arg353 (including FXII‐R353A) still activate their natural substrates PK, FXI and FXII in the presence of anions such as polyphosphate. The rates of activation for sc‐FXII‐mediated reactions are orders of magnitude lower than for FXIIa‐mediated reactions, implying that the biological activities of FXII are mediated primarily through α‐FXIIa. We suspect that the activity intrinsic to sc‐FXII serves as a trigger for surface‐dependent conversion of FXII to α‐FXIIa, with α‐FXIIa then producing physiologic effects.

**Figure 4:**
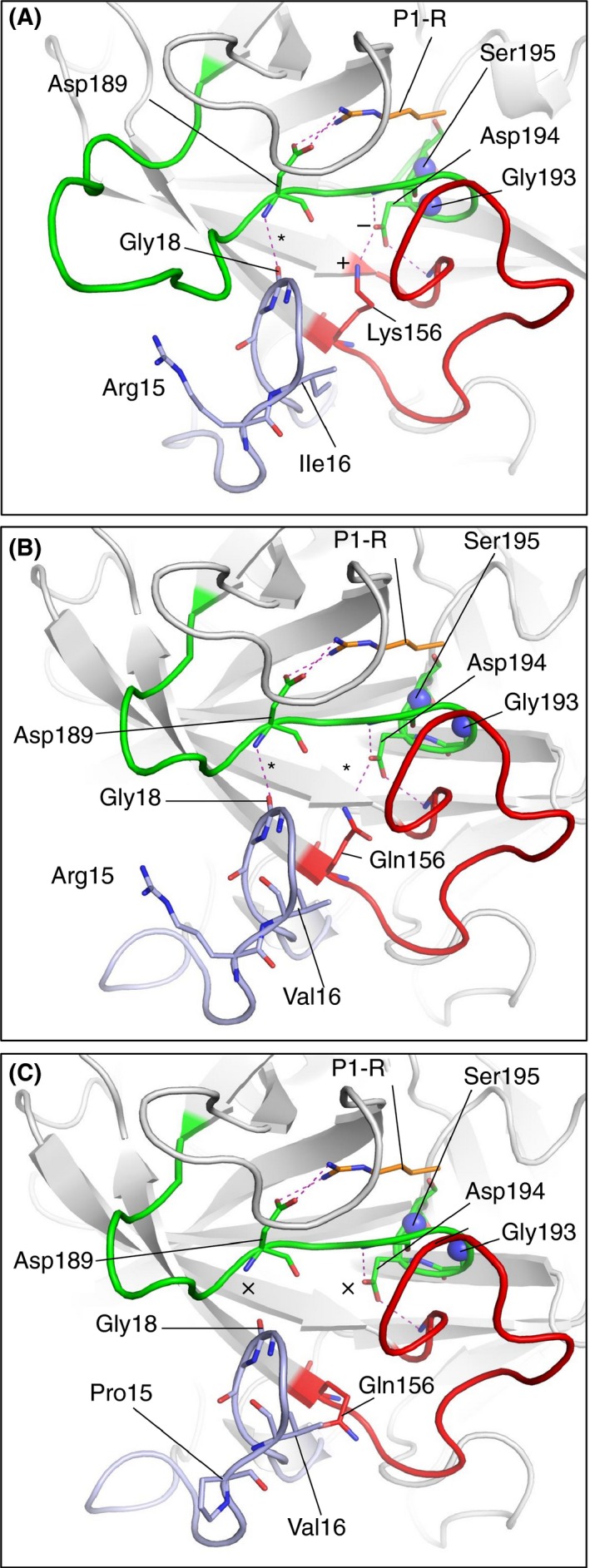
sc‐tPA and sc‐FXII structures. Ribbon diagrams show the relationship of the activation loop to the catalytic domain for (A) tPA and (B) FXII‐WT and (C) FXII Locarno. Hydrogen bonds and electrostatic interactions are indicated as dotted lines (purple). Specific residues are indicated using the chymotrypsin numbering system. Activation loops are shown in light blue, 140‐loops in red, and 180‐loops in green. The position of the oxyanion hole is indicated by the juxtaposed blue spheres that represent the nitrogen atoms of Gly193 and Ser195. (A) Single‐chain tPA structure (pdb:1BDA) shown with Asp194 stabilized by a salt bridge formed by Lys156 on the 140‐loop (Indicated by + and − symbols). Gly18 on the activation loop forms a hydrogen bond with Asp189 in the 180‐loop (*). The yellow stick figure represents the side‐chain of the P1 arginine of the inhibitor dansyl‐Glu‐Gly‐Arg‐chloromethylketone. (B) Homology model (SWISS‐MODEL 19) of FXII based on the tPA structure. As in tPA, Gly18 forms a hydrogen bond (*) with Asp189. Gln156 on the 140‐loop forms a hydrogen bond (*) with the Asp194 carboxylate group. The yellow side‐chain is the P1 arginine of a substrate (PK or FXI). (C) Predicted structure for FXII‐Locarno. Pro15 (chymotrypsin numbering, Pro353 in FXII) alters interactions between the activation loop and 180‐ and 140‐loops, disrupting hydrogen bonds with Asp189 and Asp194 (indicated by “X”). Figures prepared with PyMOL Molecular Graphics System, Version 1.8 (Schrödinger, LLC, New York, NY, USA)

Our data show that FXII‐R353P has reduced activity compared to FXII‐R353A, indicating sc‐FXII activity is sensitive to changes in the conformation of the activation loop on which residue 353 resides. The FXII catalytic domain is homologous to that of tissue plasminogen activator (tPA), a protease that expresses substantial activity in its single‐chain form (sc‐tPA).^17^, ^18^ To compare FXII and tPA, we use the numbering system for chymotrypsin in which the activation cleavage site arginine (Arg353 in FXII, Arg275 in tPA,) is designated residue 15. In most trypsin‐like proteases the activation loop is relatively unstructured. However, in the sc‐tPa structure the activation loop is well‐defined, forming interactions with components of the catalytic domain around the S1 pocket.^18^ Figure 4A shows these interactions, with the activation loop (light blue) packing against the 180‐loop (green) and 140‐loop (red). Residues Ile16 to Gly18 form a β‐turn, with Gly18 forming a hydrogen bond with Asp189. Lys156 at the 140‐loop C‐terminus forms a salt bridge with Asp194 that is thought to stabilize the S1 pocket.^18^ Figure 4B shows a homology model based on the sc‐tPA structure that suggests similar interactions in sc‐FXII. Val16‐Gly18 forms a β‐turn in the activation loop. FXII Gln156 would not form a salt bridge with Asp194 in the same manner as Lys156 in sc‐tPA, but could form a hydrogen bond that provides some stability to the S1 pocket. The bulky Pro353 in FXII Locarno is predicted to induce curvature in the main chain due to preference for discrete torsion angles (Figure 4C). Increased rigidity in the activation loop would likely disrupt the interaction with the 180‐loop and reposition the Gln156 side chain so that it no longer interacts with Asp194. These changes are consistent with reduced activity in FXII‐R353P relative to FXII‐R353A, and support the hypothesis that the activation loop of FXII, similar to the activation loop of sc‐tPA, contributes to stabilization of an active conformation.

## AUTHOR CONTRIBUTION

B. M. Mohammed performed experiments on the activity of FXII‐R353P, prepared FXII‐R353P recombinant protein, and wrote the manuscript. I. Ivanov performed chromogenic assay of FXII variants with polyphosphate, prepared FXII‐R353A recombinant protein. A. Matafonov performed the thrombin generation assays. J. Emsley performed homology modeling of FXII. D. Gailani was responsible for oversight of the project and preparation of the manuscript.

## RELATIONSHIP DISCLOSURE

None of the authors have any disclosures relevant to this paper.
